# Psychometric evaluation of a radio electric auricular treatment for stress related disorders: a double-blinded, placebo-controlled controlled pilot study

**DOI:** 10.1186/1477-7525-8-31

**Published:** 2010-03-20

**Authors:** Salvatore Rinaldi, Vania Fontani, Lucia Aravagli, Piero Mannu

**Affiliations:** 1 Medical School of Occupational Medicine, University of Florence, Italy; 2 Rinaldi Fontani Institute, Florence, Italy

## Abstract

**Background:**

The aim of this double-blind randomized study is to test the efficacy of a radio electric stimulator device using an auricular reflex therapy protocol for stress-related symptoms.

**Methods:**

The study has been carried out on 200 subjects (138 females, 62 males) that voluntarily came to our Institute declaring to "feel stressed".

The participants were randomly allocated with a computerized procedure: 150 were treated with auricular therapeutic protocol with radio electric stimulator device (REAC) and 50 were treated with an inactivated, placebo REAC. Psychological stress was evaluated trough the self-administered questionnaire Psychological Stress Measure (PSM). Assessment data were collected at 2 time points: before the treatment (T0) and immediately after the therapy cycle of 18 sessions about 4 weeks later (T1).

**Results:**

In the group treated with REAC, the psychometric evaluation after the therapy's cycle showed a significant reduction of PSM total scores, from 107.8 ± 23,13 at T0 to 87.1 ± 16,21 at T1 (p < 0.5), while in the control group no significant variation in decreasing stress-related symptomatology has been noted (107.86 ± 25,80 at T0 and 106.32 ± 25,88 at T1 (p = NS).

**Conclusions:**

The protocol of the auricular treatment with REAC seems to reduce the subjective perception of stress, as "psychometrically" demonstrated by the significant reduction in PSM test total score. This therapeutical procedure also provides a non invasive, not painful and very simple innovative approach to treat the widely diffused stress related disorders.

**Trial Registration:**

This trial has been registered in the Australian New Zealand Clinical Trials Registry (ANZCTR) with the number: ACTRN12607000529448

## Background

Environmental stress produces important effects on the individual health. Chronic social stress is one of the most important factors [1-3] responsible for worsening stress related symptoms [4,5] and for triggering previously undetected mood disorders [6,7]. In recent years, the effects of social stress on psychopathologies development have been thoroughly investigated [4,6,8,9]. It has been hypothesized that life stress alters the dynamic regulation of the autonomic, neuroendocrine [10] and immune systems [11].

The Central Nervous System (CNS), as the main biological target of stressful events [2,7], has continuously to change to accommodate to rapid environmental development. This imperceptible adaptation process is detrimental to health and quality of life [12].

Allostasis represents the adaptation process of the complex physiological system to physical, psychosocial and environmental changes or social stress [13-15]. The term "allostatic load" was coined by McEwen and refers to physiological costs of chronic exposure to neural or neuroendocrine stress response [16,17]. The allostatic state is unable to guarantee the good management of the physiological systems and consequently the health status and the individual's well-being, and this phenomena often leads to the development of psychological and psychiatric symptoms [18-32]. Among the several non-pharmacological strategies today available to reduce and/or to treat the symptomatic effects of the stress and to improve the allostatic response [33,34], of particular interest appears the use of electrical and electromagnetic stimulation [35], also exploiting the "classical" acupuncture points [36,37]. The REAC uses a new kind of non invasive and not painful electromagnetic stimulation that produces a weak radio frequency current (RFC) easily applied with a probe to specific somatic reflex points [38].

### Aim of the Study

The purpose of this study is to verify if the use of REAC could reduce the subjective perception of stress and stress-related symptoms, evaluated by PSM at T0 and T1 [39-41]. PSM also allows the precise classification of the subject studied on a stress well-being scale, to accurately assess the effectiveness of the treatment.

## Materials and methods

The study was performed in accordance with the Declaration of Helsinki, with the Societa di Ottimizzazione Neuro Psico Fisica e CRM Terapia institutional ethics committee approval, and all subjects provided written informed consent. A double-blind, randomized and placebo-controlled trial was performed to investigate the effectiveness of REAC auricular treatment in reducing of self perception of stress and stress-related symptoms and, consequently, the PSM total score.

### Participants

The study has been carried out on 200 subjects (138 females, 62 males), that voluntarily came to our Institute suffering from a broad stress-related range of psychological symptoms such as affective-emotional reactions i.e. sadness, irritability, anger, depression, poor concentration and attention, insecurity, anxious and apprehensive condition, depression, sleep disorders, generalized muscle tension, gastro intestinal disorders, headaches. Participants were properly informed about significance and objectives of the study and then they completed, in association with the Project Director prior to enrolment, a written consent form. For all participants were properly collected and detailed: general demographic information, background characteristics, treatments history, drug history survey and medical examination.

The participants, selected after a clinical screening, have been allocated randomly into 2 socially and demographically matched groups with a computerized procedure: Group A, treated with "active" REAC (N = 150, 46 males, age = 49.8 ± 13.7 yrs and 104 females, age = 48.3 ± 12.5 yrs), and Group B, treated with deactivated-placebo REAC (N = 50, 16 males, age = 52.7 ± 17.7 yrs, and 34 females, age = 50.6 ± 15.1 yrs). The 3:1 proportion between cases and controls has been selected only on the base of the main "typology" of the people who require a clinical treatment in our Institute: "stressed" subjects are more numerous than "non-stressed" subjects.

REAC protocol consists in 18 session each one lasting 3,5 seconds, administered in about 4 weeks.

All subjects were over 18 years old, declaring to suffer from stress-correlated psycho-physical conditions, as demonstrated by a clinical evaluation and the PSM total score >45. No history of drug abuse, nor severe mental illness or personality disorder according to DSM-IVTR criteria (as valuated by a psychiatrist, also trough the administration of SCL-90 scale in the "doubt" subjects) and no psychopharmacological-psychological interventions has been reported.

Exclusion criteria for both groups: diagnosis of axis I-II psychiatric disorders, organic CNS pathology, preceding skull traumas with loss of conscience longer than 5 minutes, dementia, alcohol dependence, current therapy with medications known to affect cognitive functioning.

### Randomization

The study has been setted in 4 rooms. In 3 of the 4 rooms has been activate REAC and in the other 1 the placebo-REAC. The physicians that have participated in the treatment's administration turned in the different rooms, using both "active" and "inactive" REAC but they didn't know if they used an active or placebo REAC. The computerized randomization process, managed by an external operator to the study, foresaw the assignment of the subjects to each room. The target sample size 200, divided in the 4 rooms, has produced 4 subgroups, each composed by 50 people (n = 50 × 3 = 150 treated, n = 50 × 1 = 50 placebo).

### Instruments and materials

#### Psychological Test

PSM, a standardized and validated self-reporting test for the measurement of psychological stress was administered to all subjects, before (T0) and after REAC treatment (T1) [39-41]. PSM consists of 49 items for the self-evaluation aimed at identifying different "clusters" of symptoms such as loss of control, irritability, psycho-physiological sensations, confusion, anxiety, depression, physical pain, hyperactivity and agitation. The patient must answer to the various items ranking the intensity of his psychological stress condition (very much = 4, much = 3, little = 2, none = 1). Although the clear risk of subjectivity of evaluation, this self-administered questionnaire was preferred with the aim to reduce the increasing of psychic and somatic anxiety levels and "arousal" inevitably due to a "direct" subject-physician interaction.

#### Description of the Radio Electric Asymmetric Conveyer REAC

The REAC is an innovative medical device [38] aimed to promote a reduction of the dysfunctional modifications in the Nervous System induced by stress and psychological factors [42]. REAC uses the radio electric effects produced by the interaction between the electromagnetic field of the human body (~30-300 GHz, of about 3 mW/m2) [43] and the field produced by the instrument (2.4 or 5.8 or 10.5 GHz, measurable from the emitter about 0.1 mW/m2) which lasts approximately a few milliseconds. This radio electric interaction is received by a probe (conveyer) placed on specific points of the auricular pinna giving a radio electric stimulation. The inactive REAC is the exact same machine, but has a modified probe. The instrument that we used is registered under the trademark Convogliatore di Radianza Modulante - CRM produced by ASMED, Italy.

#### Description of auricular therapy protocol

The auricular treatment protocol has been conceived by the authors and called Neuro Psycho Physical Optimization (NPPO). The REAC probe was applied to 7 specific points of the auricular pinna: "shen men", kidney, stomach, heart, occiput, ipotalamus, prefrontal cortex. Eighteen sessions of NPPO with REAC were administered on alternate days, in about four weeks, to each patient. Each therapeutic session lasted approximately 3 seconds. The protocol is painless, non-invasive, and without known side effects.

#### Statistical analysis

Statistical analysis were performed using Statistical Package for Social Science (version 13). The McNemar test is used to compare the relevant frequencies for data resulting from PSM test in patients belonging to group A and to group B before and after NPPO with REAC. Also Wilcoxon Signed Ranks Test and Sign test are used to evaluate the related samples of Total points resulting from PSM test of both groups. All results of p < 0.05 were considered statistically significant.

## Results

In both groups the total stress evaluation has been measured by PSM administered before (T0) and after a cycle of NPPO. In the group A, PSM total score progressively decreases from 107.8 ± 23.1 at T0 to 87.1 ± 16.2 at T1 (Wilcoxon Signed Rank Test Z = -9.854, p = 0.00; Sign Test Z = -10.132, p = 0.00). On the contrary, no significant difference has been noted in the group B: in fact, PSM total score ranged from 107.9 ± 25.8 at T0 to 106.3 ± 25.9 at T1 (Wilcoxon Signed Rank Test Z = -1.285, p = NS; Sign Test Z = 0.312, p = NS).

**Figure 1:**
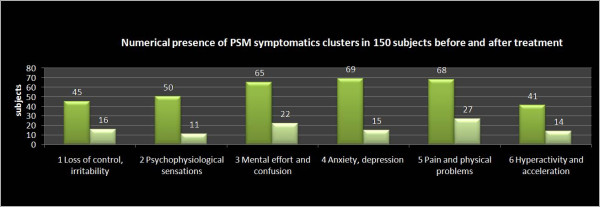
**PSM clusters pre-post NPPO-REAC real treatment, group A**. The results show that a cycle of NPPO protocol with REAC reduces also the PSM symptomatic clusters related to loss of control, irritability, psycho physiological sensations, confusion, anxiety and depression, physical pain, hyperactivity and acceleration,

**Figure 2:**
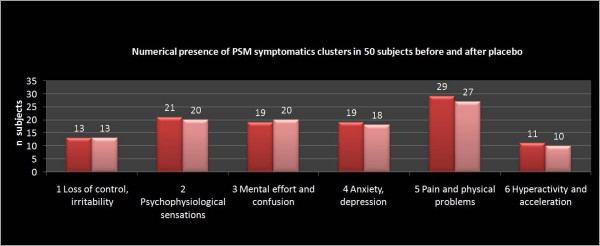
**PSM clusters pre-post NPPO-REAC placebo treatment, group B**. Group B show very similar results before and after a cycle of NPPO protocol with an inactivated REAC (placebo)

In the Group A, the greater improvement between T0 and T1 has been reported on PSM items loss of control, irritability, psycho-physiological sensations, confusion, anxiety, depression, physical pain, hyperactivity and agitation (table 1 and figure 1, 2).

**Table 1:** PSM Clusters population in group A and B before and after therapy vs. placebo

PSM Clusters	therapy (n = 150) ---- placebo (n = 50)			
	Before	After	Before	After
Loss of control and irritability	n = 45	n = 16 p < .05	n = 14	n = 13 p > .05
Psycho Physiological sensation	n = 50	n = 11 p < .05	n = 22	n = 20 p > .05
Effort sensation and confusion	n = 65	n = 22 p < .05	n = 20	n = 20 p > .05
Anxiety and Depression	n = 69	n = 15 p < .05	n = 20	n = 18 p > .05
Pain and Physical trouble	n = 68	n = 27 p < .05	n = 30	n = 27 p > .05
Hyperactivity and acceleration	n = 27	n = 14 p < .05	n = 12	n = 10 p > .05

Group A show a significantly lower presence of symptomatic clusters after the treatment with p < .05, while Group B show very similar results before and after treatment p > .05

## Discussion

The use of electricity and magnetic fields in the biomedical studies, and in particular in the treatment of disturbances of the nervous system, is not a new idea [44-47]. Nevertheless modern technology and advanced knowledge in the physical-medical field and in the neurosciences has allowed realizing the new biomedical instrument presented in this study.

**Figure 3:**
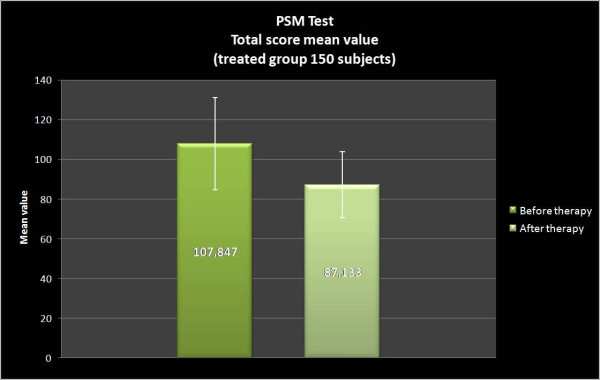
**PSM Total score mean value, treated group A**. Group A which scored 107.8 ± 23,13 (results are presented as Mean +/- S.D.) show a significantly lower score of 87.1 ± 16,21 after NPPO-REAC treatment.

**Figure 4:**
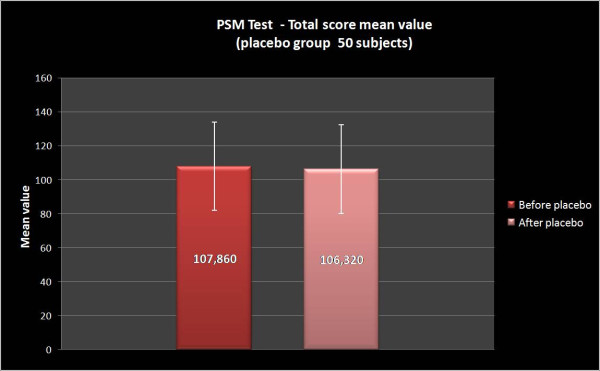
**PSM Total score mean value, placebo group B**. Group B scored 107.86 ± 25,80 (results are presented as Mean +/- S.D.) before treatment and these values were similar (106.32 ± 25,88509) after treatment.

In the present study we considered a group of patients treated with REAC and a group with placebo-REAC. The results suggest that a cycle of NPPO protocol with REAC reduces the total score of the PSM test (Figure 3, 4) in particular the clusters related to loss of control, irritability, psycho-physiological sensations, confusion, anxiety and depression, physical pain, hyperactivity and agitation (figure 1, 2), and this strictly correlates with a clinically significant improvement on the subjective perception of stress and stress related symptomatology. On the other hand, in the placebo-REAC, although the subjects have been submitted to the same "tactile" sensations on the ear of those with "active" REAC, no significant difference in PSM total score was highlighted between T0 and T1.

A growing number of studies suggests that the stress-related symptoms are the result of allostatic processes [12-15,17-19,21-23,25-27,29-31] and our data seems to indicate that NPPO protocol of REAC treatment is an effective instrument to ameliorate the responses to allostatic state and to environmental stressors.

Besides, this treatment has the advantage of being painless, non-invasive and free of side-effects not only in the patient groups, but also in the physicians that have applied this therapy.

The 3:1 proportion between case and controls certainly represent a bias of this study, mainly in terms of statistics. However, this has been simply due to the fact that our Institute is well-known with regard the treatment of psycho-physical stress-related dysfunctions and, paradoxically, it is been easier to enlist "stressed" than normal subjects. According to us, the physician's rotation in the different rooms and the fact that each one of them didn't known if were administering the "active" or "placebo" REAC, represent a attempt to achieve more "scientific" strictness.

## Conclusions

We propose that NPPO treatment with REAC will help to improve the physiological capability of the organism's recovery, optimizing the adaptive response to environmental stressors.

Moreover, NPPO treatment with REAC is a non-pharmacological treatment and can represent an efficient support in many medical fields, because it does not interfere with the simultaneous use of other therapeutic approaches.

Obviously, though these observations on the effectiveness of NPPO - REAC treatment in the psychometric evaluation of stress related disorders are certainly important and suggestive, they are only preliminary and require further confirmation.

## Competing interests

SR and VF are the inventors of the Radio electric Asymmetric Conveyer.

## Authors' contributions

SR and VF conceived of the study, participated in its design and coordination and in drafting of the manuscript. LA has critically revised the manuscript and PM has done the psychiatric evaluation. All authors read and approved the final manuscript.
